# The Effectiveness of Web-Based Psychotherapy to Treat and Prevent Burnout: Controlled Trial

**DOI:** 10.2196/39129

**Published:** 2022-08-11

**Authors:** Clive Michelsen, Anette Kjellgren

**Affiliations:** 1 Science College University of Karlstad Malmö Sweden; 2 Psychology Department University of Karlstad Karlstad Sweden

**Keywords:** risk for burnout, effectivity of web-based therapy, Proactive Occupational Health, presenteeism, work-related stress

## Abstract

**Background:**

Burnout is a hidden productivity killer in organizations. Finding a solution to efficiently measure and proactively prevent or rehabilitate employees with burnout is a challenge. To meet this unabated demand, companies and caregivers can focus on proactive measures to prevent “Burnout as an Occupational Phenomenon.”

**Objective:**

We aimed to address effectiveness, reliability, and validity of the empowerment for participation (EFP) batch of assessments to measure burnout risk in relation to the efficacy of web-based interventions using cognitive behavioral therapy (CBT) and floating to improve mental health and well-being. We introduced three risk assessments: risk for burnout, risk of anxiety, and risk for depression.

**Methods:**

We used an interventional, empirical, and parallel design using raw EFP psychometric data to measure the effectiveness of web-based therapy to reduce the risk of burnout between a control group and web-based therapy group. A total of 50 participants were selected. The rehabilitation and control groups consisted of 25 normally distributed employees each. The rehabilitation group received therapy, whereas the control group had not yet received any form of therapy. IBM SPSS was used to analyze the data collected, and a repeated measures ANOVA, an analysis of covariance, a discriminant analysis, and a construct validity analysis were used to test for reliability and validity. The group was selected from a list of employees within the My-E-Health ecosystem who showed a moderate or high risk for burnout. All assessments and mixed-method CBT were web-based, and floating was conducted at designated locations. The complete EFP assessment was integrated into a digital ecosystem designed for this purpose and therapy, offering a secure and encrypted ecosystem.

**Results:**

There was a statistically significant difference between pre- and postassessment scores for burnout. The reliability of the burnout measure was good (Cronbach α=.858; mean 1.826, SD 3.008; Cohen *d*=0.607; *P*<.001) with a high validity of 0.9420. A paired samples 2-tailed test showed a good *t* score of 4.292 and *P*<.001, with a good effect size, Cohen *d*=0.607. Web-based therapy reduced the risk for burnout in participants compared with the control group. Tests of between-subject effects show *F*=16.964, a significant difference between the control group and the web-based therapy group: *P*<.001, with movement between the group variables of 0.261 or 26.1% for the dependent variable.

**Conclusions:**

This study suggests good reliability and validity of using web-based interventional mixed methods CBT to reduce the risk of burnout. The EFP batch of web-based assessments could reliably identify morbidity risk levels and successfully measure clinical interventions and rehabilitation with consistently reliable results to serve as both a diagnostic and therapeutic tool worthy of major research in the future.

**Trial Registration:**

ClinicalTrials.gov NCT05343208; https://clinicaltrials.gov/ct2/show/NCT05343208

## Introduction

### Measuring Burnout

Burnout within the workplace is a major problem in recent times; therefore, this paper aimed to show that early web-based identification and intervention are effective in addressing this demand. Bucci and Berry [1] argued that the digital revolution is evolving at an unstoppable pace. They also accept that the explosion of digital technology and mental health care is under greater pressure to path digitally mediated interventions and services. Nonetheless, Hollis et al [2] believe that “digital technology has the potential to transform mental health care by connecting patients, services and health data in new ways. Digital web-based applications can offer patients greater access to information and services and enhance clinical management and early intervention through access to real-time patient data.” However, both Hollis et al [2] and Buccii and Berry [1] agree that there are substantial gaps in the evidence base underlying these technologies.

A meta-analysis carried out by Duijts et al [3] showed that predictors of sickness can be used in a homogeneous manner to identify presenteeism. Presenteeism relates to those who choose to remain at work, even though they are not physically or psychologically well, thereby impacting productivity, team dynamics, workflow, and the bottom line [4,5]. A literature review also revealed that key work factors associated with psychological ill-health and absence in staff were associated with long work hours, work overload and pressure, lack of control, lack of participation in decision-making, poor social support, and unclear management and work roles [4,5].

Michie and Williams [6] showed that sickness absence was associated with a poor management style. In the same report, they also showed that successful interventions used training and organizational approaches to increase participation in decision-making, problem solving, and communication techniques, as well as additional support and feedback for individuals. They concluded that many of the work-related variables associated with high levels of psychological ill-health were potentially amenable to change.

Smith and Beaton [7] raised the need for an accurate assessment of psychosocial changes in working conditions [7]. They also stressed the need for these measurements to focus on the collection of data at baseline, and if possible, a follow-up time point.

Over the years, many complex attempts have been made to measure burnout. These include, but are not limited to, assessments for job satisfaction and performance in 1991 [8], development of a measure of workplace deviance in 2000 [9], the World Health Organization’s Health and Work Performance Questionnaire in 2003 [10], the Stanford Presenteeism Scale in 2005 [11], the Utrecht Work Engagement Scale in 2002 [12], and others. What is evident and common in all the above measures is that they are static and retroactive in nature; they lack continuity in understanding an individual over time and within their changing environments. According to Michelsen [13,14], a reliable and valid methodology to support individual psychological safety and engagement needs continuity in its construct. He suggests that individual measures need to have the ability to monitor individuals within their environment, over time, and dynamically show deviations, mean scores, and trends covering a broad spectrum of issues that affect psychological well-being, engagement, and participation in a myriad of situations within the workplace.

Originally designed in early 2001, as a psychosocial monitoring tool used to assess employees undergoing transformational change initiatives within their companies [13], the empowerment for participation (EFP) battery used today has seen numerous upgrades, from its original paper format to a digital (HTML) version in 2007, to the fully interactive web-based ecosystem used currently. The assessments were accessed via a secure and encrypted web-based ecosystem.

The *EFP assessment battery* consists of 4 assessments and a 360° mirror feedback assessment when objective feedback is required (not addressed herein). All assessments are scored using the visual analog scale (VAS), which consists of a straight line with a beginning and end point; for example, very good to very poor. As the slider moves from left to right, the text positioned at either end of the line increases as the opposite end decreases. The position at which the slider stops is represented by a number from 0 to 20. The slider can be moved in either direction or in accordance with the assessed feels regarding that specific question. To minimize the clustering of points around a preferred numeric value or description as used by Likert-type assessment scales, no visible numeric values or intermediate points are visible or seen by the assessed. The *Journal of Behavior Therapy and Experimental Psychiatry* showed that the VAS scales used enable simple and rapid assessment of the state of anxiety and exhibit superior psychometric properties [15].

The EFP *Motivation Assessment* consists of 20 VAS questions (Cronbach α=.939) and aims to show the level at which the individual’s motivation lies. It measures an individual’s motivation in areas such as transparency (openness), meaning, identification, balance, teamwork, stimulation, self-esteem, participation, and engagement among others. Validation analysis showed that 95.9% of the original grouped cases were correctly classified, 93.2% of cross-validated grouped cases were correctly classified (effect size=0.881; Wilks lambda=0.106; *P*<.001), and good group centroid separations from low motivation to high motivation were noted, indicating an excellent measure [14].

The EFP *Stress Assessment* consists of 20 VAS questions (Cronbach α=.887) and aims to show the level of an individual’s stress level by measuring several stressors and symptoms in areas such as communication, knowledge, conflict, justice, values, safety, and health in the workplace. Earlier analysis showed good validity, where 91.8% of the original grouped cases were correctly classified, 89% of cross-validated grouped cases were correctly classified (effect size=0.913; Wilks lambda=0.072; *P*<.001), and good group centroid separations from low stress to highly stressed were noted, indicating an excellent measure [14].

The EFP *Defense Routines Assessment* consists of 20 VAS questions (Cronbach α=.870). The purpose here is to map and understand an individual’s defense routines and the ability to engage in work and life effectively and openly. The concept of defense mechanism was driven by Freud [16] in 1923, where more difficult-to-handle associations are considered to exist in the so-called primary process (subconscious process), usually considered as an antagonistic relation to the secondary process (conscious processes). Rather, the *Defense Routines Assessment* uses redefinition by Neisser [17] of these processes, where they are not perceived as antagonistic but instead as essential to each other. This suggests that the secondary process provides opportunities to process primary material through an appreciation of the routines that the individual creates as a defense. According to Senge [18] and Michelsen [13], defense routines are habits created to protect us from threats to our self-image and identity. Therefore, these routines provide protection against our deepest assumptions. The *Defense Routines Assessment* aims to measure these routines in areas such as guilt, flexibility, forgiveness, communication, conflict, control, and rationality. Michelsen [13] showed that 97.3% of the original grouped cases were correctly classified, 94.5% of cross-validated grouped cases were correctly classified (effect size=0.891; Wilks lambda=0.103; *P*<.001), and good group centroid separations from low defense routes to moderate defense routes were noted, indicating a very good measure [14].

The EFP *Perpetual Motivation Positioning* assessment consists of 50 VAS questions (Cronbach α=.944), divided into seven subscales: (1) knowledge, (2) external demands, (3) social environment, (4) health and safety, (5) self-expectations, (6) openness, and (7) self-esteem. The assessment is presented in a visual spider-gram for easy visual identification of areas in conflict. For example, deviations or anomalies with external demands, self-expectations, and social environments are usually associated with those persons exhibiting psychological challenges or some degree of burnout. The purpose of the assessment is to gain insight into a person’s boundaries, participation and adaptability, trust, creativity, openness, and more. In an earlier analysis of the EFP assessment, statistical validation showed that 100% of the original grouped cases were correctly classified, 97.3% of cross-validated grouped cases were correctly classified with an effect size of 0.908 (Wilks lambda=0.084; *P*<.001), and good group centroid separations from low adaptability to high adaptability were noted, indicating an excellent measure [14].

The *Risk for Burnout* report has 30 questions that make up the risk assessment (Cronbach α=.928; *P*<.001 partial eta squared=0.900; Cohen *d*=0.900). They were compiled from the EFP batch of questions as follows: (5) motivation; (5) stress; (5) defense routines; (0) knowledge; (6) *Perpetual Motivation Positioning* external demands; (0) social environment; (2) health and safety; (3) self-expectancy; (0) openness; and (2) self-esteem. There are four subdimension measures: (1) external demands, (2) self-expectancy, (3) depersonalization, and (4) symptoms. All questions are VAS formatted with values ranging from 0 to 20 and a total accumulated score ranging from 0 to 600. The purpose of the risk assessment scale is directly associated with preventive care and proactive cognitive behavior therapy (CBT), used to address any identified trends or associated risks. Because My-E-Health is a registered company health care provider, this EFP Risk for Burnout (EFPRB) measure has served and proven its reliability and validity in preventing workplace-related psychological ill-health, indicating an excellent measure [14].

To avoid psychological and central tendencies and to increase the reliability and validity of the burnout measure, several measures were taken during the year. First, individuals taking the assessments are not limited to symptomatically related questions for specific diagnoses; rather, they take holistic and situation-based questions regarding their general environmental well-being and how they feel within that context. Second, all questions are VASs, and lastly, there are embedded control questions to check for similar question variance anomalies.

There are five risk levels of burnout in the EFPRB assessment—risk levels are provided in relation to the total score or the mean score following a linear guide: 0 to 99 points (mean 0-3.300)=no evidence of burnout, 100 to 199 points (mean 3.333-6.6333)=low risk for burnout, 200 to 299 points (mean 6.6667-9.9667)=moderate risk for burnout, 300 to 399 points (mean 10.00-13.300)=high risk for burnout, and 400 to 600 points (mean 13.3333-20)=burnout. These have proven to be statistically significant in previous studies (N=73)—risk for burnout: Cronbach α=.928; *P*<.001; partial eta squared=0.900, Cohen *d*=0.900 (Michelsen, 2021 [4]). It has excellent content validity: internal validity of *P*>.93 and external validity of *P*>.91 [4].

### Prevention or Rehabilitation Interventions

Measuring and identifying burnout risks and implementing preventive measures early are simpler than retroactive approaches. Retroactive or burned-out cases are those with a high degree of causal and symptomatic reactions already embedded into one’s behavior and specifically related to their individual burnout. This requires immediate support for staff-off or sick leave. In some cases, up to 2 years. Understanding these nuances is just part of the complex equation used to evaluate the causes leading to mental health exhaustion and their symptomatic effects on the individual [4].

Internet-based therapy using CBT is now considered an effective way to treat a range of psychological disorders [19,20]. Michelsen [4] suggested that a solution can be found by merging a measurement, causal and symptomatic identification instrument, and an interactive web-based solution to improve access and efficacy in treatment.

Naturally, efficacy in treatment requires many approaches to be discussed herein, namely accessibility, psychological safety (both psychological and data-driven), chemistry (patient and caregiver), an interactive web-based environment to enhance cognitive awareness of self, and a measurement system to demonstrate patient and counselor treatment progress and empowerment.

Web-based (24/7) access provides the employee with a patient-centric ecosystem with access to a multimodal caregiver team (various specialties from a health coach, psychotherapist, psychologist, medical doctor, counselor, dietician, and others), where the individual can and even choose their care team from available caregivers. The My-E-Health ecosystem provides this type of multimodal access, and it opens the caregiver team’s booking calendar to the patient so that the patient can easily book appointments as needed.

Psychological safety should be provided for both the individual and the health care teams. According to a study by Grailey et al, in 2021 [21], a health care team requires a shared belief of psychological safety to allow for interpersonal risk taking, improve innovation, and reduce errors through team InterVision. The My-E-Health model conducts weekly InterVision. Therefore, a multimodal web-based health care team can provide psychological safety via team connectedness designed around a consensus-driven patient-centric system used by My-E-Health. In 2021, Hunt et al [22] showed that psychological safety is an important component of safe and effective patient care in mental health services. Patient psychological safety is as important as the outcome of the health care team. According to Fathers and Stevens [23], it is important to make a patient’s experience positive. They also believe that good communication (combining visual, verbal, and written communication to best convey the treatment message) is of greatest importance as well as accessible and correct information and that caregivers should speak clearly not rushing the patient but adapting to the patient’s pace. Maintaining a patient’s dignity, integrity, and privacy is a vital component of psychological safety [23]. Psychological safety includes the use of a secure and encrypted web-based framework in which secure conversation tunnels can be built between the patient and caregivers. Understanding that one’s personal data are always kept private and not shared with persons outside of the care team also supports this. Aggregate data, quality assurance, and oversight are fundamentally important for all stakeholders. This is also supported by InterVision and consensus among the multimodal teams in an internal weekly session.

According to Laughton-Brown [24], chemistry between counselors (any member of the health care team) and employees (patients) is of primary concern, as a trusting relationship is fundamental to good therapy. Facilitating this chemistry can be enhanced by the psychological safety offered by the multimodal care team. This can be improved during the initial web-based therapy (providing feedback on the results of the assessments) through cognition by connecting the visual cortex (visually recognizing and concurring with one’s status) and accepting it by validating how the patient feels. This content validation uses the cognitive dissonance theory to connect the subjective situational environment (experience by the patient) to their status and well-being.

Therapy and sick leave are recommended for individuals assessed to be “burned-out,” and they are immediately placed into the company rehabilitation program. Employees fluctuating between the lower two categories on the EFPRB scale (no evidence and low risk) require no additional care and continue their normal quarterly feedback sessions with their health coaches. However, individuals that show a “*Moderate Risk for Burnout*” level enter a modified web-based rehabilitation program to address identified concerns and receive the necessary guidance to build the needed coping skills to address said issues. Employees falling into the “*High Risk for Burnout*” category enter an individually designed intervention or proactive rehabilitation program, usually all 3 of the components described herein.

### Interactive and Integrated Web-Based Environment

The advantage of the web-based environment over that of traditional clinical settings is that it can be interactive and integrate a myriad of digital and visual tools and various media. Marikyan et al [25] also believe that connecting the dots using cognitive dissonance in technology adoption is rapidly becoming the norm. For web-based therapy systems to be as effective as possible, an interactive ecosystem that provides users with the ability to adapt treatment models to the needs of the patient, and not the reverse. Furthermore, patient-validated assessments can monitor progress if assessed monthly during treatment. Michelsen [4] believes that visual and cognitive recognition and interactivity among the patient, caregiver, and interactive environment help empower the individual [4].

Web-based tools can be used to enhance clinical therapy sessions by providing interactive solutions, as used in Gestalt therapy, to demonstrate possible patient cognitive distortions, dissonance, and self-awareness and visually illuminate areas that may inhibit self-empowerment. These tools can be used in one or a combination of various psychological disciplines (CBT, acceptance commitment theory, and dialectical behavioral therapy and psychodynamic, interpersonal, and transpersonal) and can transform how future web-based mental health ecosystems can work. Finding a workable and sustainable solution that can proactively monitor and positively impact mental health and the effects of burnout will ultimately support systemic evidence-based interventions to identify and manage presenteeism [14].

## Methods

### Recruitment of Participants

The participants were employees of contracted companies within the My-E-Health company health care and well-being platform. Both the control and therapy groups were gainfully employed and belonged to the My-E-Health company health care program. Their ages ranged from 22 to 68 (mean 43.28, SD 11.317 years) with homogeneity. The study consisted of 28 (56%) males and 22 (44%) females with a mean of 1.44 and SD of 0.501, suggesting good homogeneity. At the time of their assessment, employees had been working at specific positions for 12 months to 16 years.

The control group (n=25, age: mean 42.92, median 41.00, SD 10.71 years) consisted of 11 men (44%) and 14 women (56%). The treatment group (n=25, age: mean 43.64, median 41.00, SD 12.10 years) consisted of 17 (68%) men and 8 (32%) women.

### This Study

The aim of this study was to evaluate the effectiveness of web-based therapy in addressing and lowering the risk of employee burnout among randomly selected employees in 4 private companies located in Sweden and the United Kingdom. The hypothesis is that, compared with the control group, the intervention group will demonstrate decreased levels of burnout and that these improvements will remain after 6 months. The research questions of this study were as follows:

1. Is web-based therapy effective in reducing existing burnout?
2. Is web-based therapy effective in reducing the risk for burnout for participants compared with the control group?

### Clinical Trial Design and Criteria

This study was approved by the Regional Ethics Committee in Lund, Sweden, in 2017 and began before clinical trial registration. This interventional and empirical study examined the effectiveness of web-based CBT in reducing burnout. A parallel study of a control group was conducted. Both the intervention therapy group and the control groups were selected from a list of employees within the My-E-Health company health care ecosystem that had been assessed on the EFP Burnout Scale to have “Moderate Risk of Burnout,” “High Risk of Burnout” or with “Burnout.” Inclusion criteria include the following: (1) a moderate or high risk for burnout; (2) employees with a current burnout diagnosis from a hospital or outpatient or psychiatric clinic; (3) a fully employed person with a member organization; and (4) no other inclusion criteria. The exclusion criteria were (1) no evidence of burnout on the EFP scale, (2) low risk of burnout on the EFP scale, (3) unemployed persons, and (4) no other exclusion criteria. The inclusion group (n=50) consisted of a control group (n=25) and interventional therapy group (n=25).

The postassessment value was used as the dependent variable. The control group consisted of 25 normally distributed employees (n=25) who had not yet received any form of therapy but only received feedback related to their EFP assessment scores and a therapy group (n=25) consisted of those who received interventional CBT. Both groups were required to validate the pretest and posttest EFP assessment results. Repeated measures ANOVA and analysis of covariance (ANCOVA) analyses were performed using the SPSS (IBM) program.

### Intervention Package Used to Reduce Moderate and High Risk for Burnout

The therapeutic package developed by My-E-Health for the treatment of employees, health care professionals, and patients with moderate-to-high risk for burnout consists of three components:

1. The web-based test battery, known as the EFP assessment and the Risk Assessment for Mental Exhaustion (identify the risk level for burnout) and many of the causal aspects related to that risk level.
2. Web-based therapy that is either proactive or preventive in nature or retroactive and encompasses mixed-methods therapy using a mix of CBT, acceptance commitment theory, dialectical behavioral therapy, transpersonal and psychodynamic therapy, as well as mindfulness techniques such as mindfulness walks (isolating the sensors independently during a walk) and other parasympathetic bottom-up or top-down strategies. These help to bring the patient into the present while addressing both the sympathetic response (fight or flight response) and parasympathetic response (rest or relax and digest response) approaches, also complemented with daily routines to stimulate natural hormone release.
3. Flotation REST (Restricted Environmental Stimulation Technique) completed at any of many floating centers internationally, a minimum of 10 floating sessions 2 to 3 times weekly (depending on the level of exhaustion) for 4 to 5 weeks. Each floating session was followed by a web-based therapy session.

One purpose of *the first component* (EFP) is to detect the severity, early symptoms, and associated causes that lead to a risk for burnout. This facilitates preventive and proactive treatment while at work and not waiting until someone is on sick leave. Another aim was to provide an adequate description of problems (symptomatic and causal in nature), irrespective of severity, for appropriate treatment methods to be introduced and for proper monitoring and evaluation. The psychometric assessments included in the EFP represent a compilation of a range of well-tested test techniques, which are also found in other psychological tests. The EFP batch assessment uses a new approach in that it uses well-tested techniques that have been combined into a whole, where the different parts are allowed to mirror and deepen the understanding and correlation with each other to provide a better overall assessment of an individual’s psychological challenges, causes, hurdles, or conditions. Here, individuals can connect with their coach, caregiver, or multimodal team, complete their psychometrics (as needed in therapy or at least on a quarterly basis) and receive live, interactive, face-to-face feedback about their results.

*The second component*, the web-based therapy or mental health coaching program, focuses on improving the individual’s situational awareness and understanding of the present, to addressing any cognitive distortions or cognitive dissonance and empowering a path forward. This is accomplished by using an interactive web-based ecosystem as well as learning to identify and listen to their signals and using “mixed methods CBT” and mindfulness to activate their visual cortexes to empower themselves to change. The phase can also include activities designed to help individual patients listen to their bodies and nervous systems (sympathetic and parasympathetic) and to naturally manage hormonal stimulation via daily routines [26-33]. International studies show that web-based counseling combined with CBT (in all its forms) and associated decluttering activities such as mindful-walks, yogic breathing, meditation, yoga, yin yoga, yoga nidra, visualization, aroma therapy, hot shows, cold therapy, and much more, can be used effectively for a variety of clinical problems [34-37]. There are many different relaxation techniques, such as meditation, Tai-Chi, yoga, and qigong [38]; nonetheless, these methods often require long and regular practice before the benefits become apparent. Therefore, a combination of relaxation exercises to activate the parasympathetic nervous system and an effective and well-known method to reduce physiological and psychological responses to stress and anxiety [39] is needed. Reducing stress with stress-reduction therapy and relaxation training to increase a sense of “mindfulness” usually involves many hours of practice. It is also understood that people with the greatest need for relaxation training are also those having the toughest time in implementing and completing such training [40].

Posttherapy treatment includes continuity with web-based quarterly feedback, which has proven to be an effective way to solidify treatment and prevent relapse. Nonetheless, the evidence for web-based CBT and other methods is well investigated (Carlbring et al, 2018 [40]) and need not be further discussed here. For patients with a moderate-to-high risk of burnout, this phase starts and coincides with the third component.

In contrast, *the third component,* the flotation REST, may not be as well known; therefore, it will be described in more detail below. In step 2, we focus on the treatment of clinical and symptomatic parameters affecting the patient. These include interactive situational analysis of the patient in relation to their holistic self: causes and empowering solutions to empowerment and symptomatic therapy. This can include decluttering exercises to help reduce the constant onslaught of stimuli that activates the autonomic nervous system (sympathetic and parasympathetic nervous systems) and many others. It is well known that a heightened state of stimuli leads to an overactive amygdala signaling the hypothalamus and autonomic nervous to be on guard or in “Fight or Flight” mode [41]. This state increases involuntary body functions such as blood pressure, pulse, dilation of the blood vessels, and breathing caused as the adrenal glands stimulate a hormone called epinephrine (adrenaline) and secrete it into the blood [41]. Consequently, the hypothalamus and pituitary gland (HPA axis) triggers the release of blood sugar (glucose and fats) as well as the activation of various corticosteroids. This, in turn, affects an individual’s circadian rhythms, sleep, fatigue, and behavior. Patients exhibiting various degrees of stress, anxiety, depression, or burnout usually find themselves in a heightened state, an exhaustive state, or somewhere in between [42]. Step 3 flotation REST or float therapy acts as a break to the sympathetic nervous system by activating the parasympathetic nervous system (producing the rest, relax, and digestive state) by eliminating extra stimuli, muscle tension, stress, and more coming from within or from the situational environment facing the patient [43].

Flotation REST was first introduced by Dr John Lilly in 1954 as an isolation tank to help isolate persons from gravity, the onslaught of external stimuli. Experiences of Dr Lilly with weightlessness and high-altitude flight and isolation also seem to coincide with the meditative experiences of Robert A Monroe, the founder of The Monroe Institute, and his meditative experiences related to monotonous low-frequency stimuli in the exploration of human consciousness leading to a state of rest or relaxation. Both the aforementioned practiced isolation and a form of deep meditation.

Benson [44] argued that the positive effects derived from these various relaxation techniques may be due to deep relaxation, which simultaneously triggers the so-called a relaxation response (RR). Yogic breathing techniques can also improve the transition effectiveness after entering the tank to achieve a state of relaxation [45]. It is also well known that individuals in a state of acute crisis are not, as a rule, susceptible to rehabilitation efforts, so catching challenges early and combining the 3 proactive components within the intervention program where individuals can benefit from more professionally oriented interventions, creating distance and learning the necessary coping skills to manage daily challenges [45].

### Relax Response

Relax response (RR) is identified as the physiological opposite of the “fight-flight response” or stress response [46]. RR is associated with instantly occurring physiological changes, including reduced sympathetic nervous system activity; reduced metabolism; and lowered heart rate, blood pressure, and respiratory rate [45,47]. Activation of the parasympathetic nervous system deactivates corticosteroid effects and improves recovery, improves sleep quality [42], and further creates less dependency on alcohol and psychoactive medications, while increasing the sense of control and efficacy in stressful situations [48].

Currently, techniques are used to induce relaxation and trigger RR. Many symptomatic pharmacological treatments rarely succeed in successfully treating stress-related disorders [49].

Ben-Menachem [50] believed that two main factors were necessary to trigger RR: (1) reduced sensory stimulation and (2) decreased body movement. Flotation REST triggers RR (parasympathetic nervous system) by removing many of the negative stimuli by introducing effortless deep relaxation in a weightless, dark isolated floating pod [50]. It has been shown that the effect of a rehabilitation program diminishes if delayed too long. Introducing rehabilitation efforts at an earlier stage using proactive relaxation therapy, in conjunction with mixed methods CBT, for those displaying a moderate-to-high risk of stress, anxiety, or burnout [14], ensures faster recovery.

The floating pod or floating chamber was filled to a height of approximately 20 cm with lukewarm water (approximately 35 °C-36 °C or 95 °F-96.8 °F) mixed with a magnesium sulfate to water specific gravity ratio of 1.25:1. The chamber can be either a large room designed for patients with claustrophobia or a pod that resembles an egg shape. The patient literally floats like a cork in a weightless environment during their floating sessions. Some patients have likened it to the safety of a womb before birth. Some spas that use floating tanks may use soft music during a floating session; nonetheless, no music is played if one is in therapy. Tanks are equipped with soft lights; however, the patients turn them off when comfortable. Sensory isolation is achieved when decluttering emerges, and an individual finds mindfulness by intuitively and visually moving into the primary process [43]. In psychology, this is referred to as the primary process. As decluttering, breathing, and meditating take hold and the patient achieves the here-and-now or present, the secondary process emerges for a more mature style of thought. In some cases, patients may fall asleep during this phase. Professor Kjellgren has shown in her research that it is restful and psychologically beneficial to experience recurring episodes of just “being in the present weightlessness state” induced by a flotation tank [51].

The flotation tank is constructed such that the participant has full control and can stop when they wish to. The lid is easily opened from the inside as well as from the outside. By pressing the buttons inside the tank, the participant could turn on a light in the tank or call for the staff. The water salinity is remarkably high, which provides a good carrying capacity. The high buoyancy in combination with the low water level (approximately 30 cm or 12 inches in depth) provides additional psychological safety (needed for the parasympathetic nervous system to activate) and security. The water is automatically circulated and filtered through a 5-µm filter and processed via a UV light system before and between the floats. Each participant floats in a safe and clean environment. At the same time, it must be emphasized that flotation REST is a “mild” form of REST, and there are no reports in the literature on hazards or problems to patients.

Jonsson [52] showed that these techniques can trigger RRs even in patients with severe anxiety (generalized anxiety disorder), depression, posttraumatic stress disorder [52], and burnout.

### Ethics Approval

The study protocol was approved by the Regional Ethical Board in Lund, Sweden (Dnr 2017/761). All personal data were anonymized, all cases were provided with identification tags, and all individual identifying information was excluded before any research and when the data were transferred into the matrix used for statistical analyses.

### Procedures for Assessment, Collection, and Data Analyses

Data were extrapolated from the organizations in the My-E-Health program. All employees agreed to the co-ownership of the data and participated in the research. All individuals have approved our General Data Protection Regulation (GDPR) regulations and responsibilities and are currently or were members of our company health care program. A total of 73 (n=50) employees took 100 batches of EFP assessments. However, for the purpose of this study, only the first and last assessments will be used. Employees were informed about the ongoing research and the objectives of the studies, anonymity, and confidentiality of the survey.

Because all assessed individuals entering the My-E-Health EFP platform were unknown upon entry (as little information was known about them before their assessments, no prequalification or demographics were applied, and no individual categorization or adjustments were made), they should be considered one large cohort. There was only one contingency, and all assessed individuals within this study were full-time employees. Their assessments were subjective and purely based on how individuals interpreted their own situational environment, health, and well-being. Personal data were strictly kept private between the individuals and their counselors or caregivers. No information was made available to employer organizations other than the aggregate data pertaining to their company.

### Procedure, Access, Measures, and Web-Based Treatment

For web-based therapy to reach its full potential, individual integrity, privacy, solid measures with reliability and validity, and an interactive framework are necessary. Naturally, the web-based ecosystem needs to provide psychological safety where individuals can feel safe, access an encrypted “Private Space,” and receive live video feedback within an interactive environment. The EFP batch is accessed via a web-based ecosystem designed to proactively support individuals in their eHealth needs. Here, individuals can connect with their coach, caregiver, physician, or psychologist; complete their psychometrics (as needed or at least on a quarterly basis); and receive live counseling about their results. All communications were encrypted and performed within a secure (Health Insurance Portability and Accountability Act– and General Data Protection Regulation–compliant) framework to protect individual integrity. Any identified deviations can be addressed early together with the caregiver or certified counselor, using the inbuilt CBT framework.

**Figure 1:**
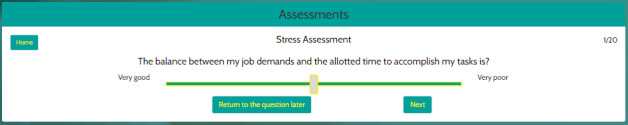
Visual analog scale used in assessments.

The EFP assessment battery consists of the 4 assessments discussed earlier. All assessments are scored using the VASs, consisting of a straight line with a beginning and end point; for example, very good to poor (Figure 1). As the slider moves from left to right, the text positioned at either end of the line increases as the opposite end decreases. The position at which the slider stops is represented by a number from 0 to 20. The slider can be moved in either direction or in accordance with the assessed feels regarding that specific question. To minimize the clustering of points around a preferred numeric value or description as used by Likert-type assessment scales, no visible numeric values or intermediate points are visible or seen by the assessed. The *Journal of Behavior Therapy and Experimental Psychiatry* showed that the VAS-A scales used below enable a simple and rapid assessment of the state of anxiety and exhibit superior psychometric properties (Abend et al, 2014 [15]).

Figure 1 shows an EFP assessment question scored using the VAS, consisting of a straight line with a beginning and end point: very good to poor in this example. The VAS line has hidden values to minimize central tendencies. The button can be moved in either direction, either left or right based-upon how an individual may feel, and the scale variables increase or decrease proportionally*.*

### Statistical Analysis Requirements

Several assumptions that needed to be met before running the analysis of the pre- and posttest data were made. The ANCOVA will control for the pretest values to analyze the posttest values and examine whether there is a significant difference between the control group and the therapy group while controlling for the pretest values. ANOVA requires an assumption of normality where both the pretest and posttest are normally distributed.

#### ANCOVA Assumptions

To run an ANCOVA, 2 assumptions must be met to ensure that the covariate meets the requirements to run the ANCOVA. The first assumption is that the pretest cannot be statistically significantly different across the levels of the independent variables of the group. (n=50; control group, n=25, and web-based therapy group, n=25). This means that there was no difference between the control group and the treatment group for the pretest or initial assessment test.

The procedure used a general linear model univariate analysis to check whether the covariates met the requirements to run the ANCOVA. Assumption test 1, testing between-subject effects using the pretest for burnout as the dependent variable, showed no significant difference between the groups for burnout (*P*>.07), thereby passing the first assumption.

The second assumption is the homogeneity of the regression test between the subjects’ effects. The dependent variable is the posttest assessment for burnout, and the covariate is the pretest assessment for burnout, where the group was the fixed factor. To pass this assumption, the group times the pretest for burnout cannot be significant. The results showed a nonsignificant result of *P*>.13, thereby meeting the homogeneity of the regression condition and passing the requirements needed to move forward with an ANCOVA.

#### ANOVA Assumptions

There are 4 assumptions that need to be met to complete a repeated measures ANOVA with normality being the most important, where both the pretest and posttest are normally distributed:

The dependent variable should be interval or ratio data.

1. The participants are randomly sampled from the population.
2. The dependent variable is normally distributed in the population at each level of the independent variables.
3. The variability in the difference between any pair of groups should be the same as that between any other pair of groups.

The test for normality showed no missing values and that the data did not exhibit statistically different normality values for both the pretest and posttest (Kolmogorov-Smirnov test=0.200), confirming the null hypothesis. Shapiro-Wilk analysis for normality for the burnout pretest (*P*=.69) and for the burnout posttest (*P*=.17) confirmed that we could. This result confirms that the null hypothesis can be accepted (or cannot be rejected).

A descriptive analysis showed acceptable burnout pretest (skewness=−0.179 and kurtosis=−0.565) and burnout posttest (skewness=0.027 and kurtosis=−1.057) values for skewness and kurtosis. The EFP pretest assessment for burnout showed a normal distribution (n=50; mean 7.62, SD 2.90). The EFP posttest assessment for burnout showed (n=50; mean 5.80, SD 2.612). There were no outliers, and both pre- and posttest EFP assessments showed a normal distribution.

Paired samples statistics showed group mean scores for the pretest for burnout at mean 7.624 with an SE of 0.41008 and posttest mean 5.7980 with an SE of 0.36946, meeting the assumption to run the ANOVA. Paired samples correlations showed no correlation, 0.408 and *P*<.003 to meet the assumption needed to run ANOVA. Therefore, all 4 assumptions for running the ANOVA were met.

Normality was not violated, and the assumptions for both the ANCOVA and ANOVA were met.

## Results

### Overview

The results are organized into four sections: an ANCOVA, a repeated measures ANOVA, reliability for internal validity using discriminant statistics, and external content validity analysis.

**Figure 2:**
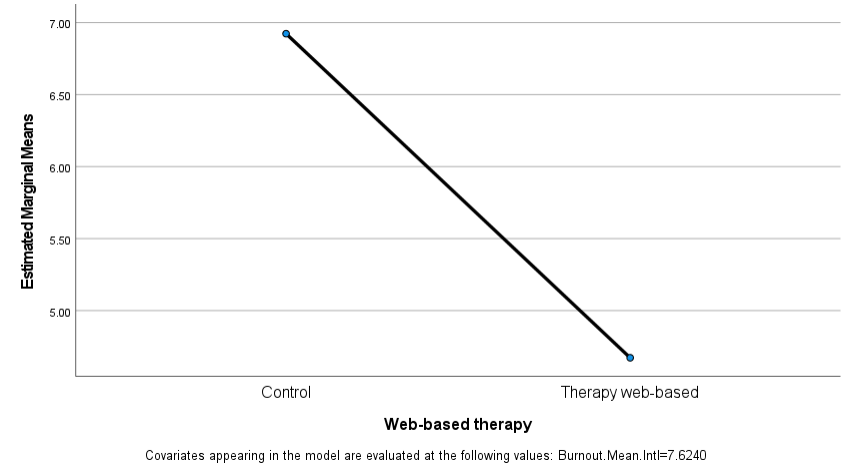
Pretest and posttest results.

A descriptive output of the ANCOVA showed a clear difference in the mean scores for the control group, mean 6.572, SD 2.54 and the web-based intervention CBT group, mean 5.024, SD 2.49 where a lower score represents a lower risk for burnout. In controlling the pretest assessment for burnout scores, the between-subject effects group analysis showed statistically significant differences between the groups on the posttest assessment for burnout: *F*=12.624, *P*<.001, and a partial eta squared (0.212) or membership difference between the groups of 21.2% (Figure 2).

Figure 2 presents the estimated marginal means for the posttest assessment for burnout between the groups, which shows a significant drop in the risk for burnout for the intervention CBT web-based therapy group as opposed to the control group.

### Repeated Measures ANOVA

When running a repeated measures ANOVA, the paired samples test showed good results (*t* score of 4.292; *P*<.001 with a good effect size Cohen *d*=0.0607) obtained from the mean average 1.826 divided by the SD 3.008. The paired samples effect sizes for the pretest (Cohen *d*=0.607, 95% CI 0.302-0.906) and posttest (Cohen *d*=0.602, 95% CI 0.300-0.899) also showed good effect sizes.

Using the general linear model ANOVA between subjects to analyze the difference between the pre- and posttest scores as a dependent variable and the n=50 group (the control and intervention CBT group) as the fixed factors, with the posttest assessment for burnout as the covariate, the descriptive statistics for the univariate analysis of variance showed a significant difference in the mean scores for the control group (mean 0.304, SD 2.085) versus the intervention CBT web-based group (mean 3.348).

The Levene test of equality of error variances tests the null hypothesis that the error variance of the dependent variable is equal across the groups. Burnout difference based on mean (Levene_1,48_=3.629; *P*=.06). Burnout difference based on median (Levene_1,48_=2.354; *P*=.13). This fails the assumption; therefore, the null hypothesis can be accepted.

Tests of between-subject effects (Table 1) show that *F*=16.964 and a significant difference between the control group and the web-based therapy group: *P*<.001 with an effect size or movement between the group variables or change of 0.261 or 26.1% for the dependent variable.

An independent-samples *t* test, with the difference in burnout scores between the groups, where the difference in score becomes the dependent variable and the group the fixed factor is presented in Table 2. Group statistics for the independent-samples *t* test using the difference in burnout scores as the dependent variable showed a large mean difference for the control group (mean 0.3040) and the web-based therapy group (mean 3.3480). This is confirmed when the equal variances assumed are calculated using the Levene test for equality of variances (*F*=3.629; significance=.063; *t*_48_ test=0-4.119; *P*<.001; CI −4.5299 to −1.5580).

Table 2 shows a clear burnout difference between the control group and the intervention group.

**Table 1:** Tests of between-subject effects shows a 26.1% difference between the groups (dependent variable: burnout difference).

Source	Type III sum of squares	*df*	Mean square	*F* value	Significance *P* value	Partial η^2^
Corrected model	115.824^a^	1	115.824	16.964	<.001	0.261
Intercept	166.714	1	166.714	24.417	<.001	0.337
Group	115.824	1	115.824	16.964	<.001	0.261
Error	327.732	48	6.828	N/A^b^	N/A	N/A
Total	610.270	50	N/A	N/A	N/A	N/A
Corrected total	443.556	49	N/A	N/A	N/A	N/A

^a^*R* squared=0.261 (adjusted *R* squared=0.246).

^b^N/A: not applicable.

**Table 2:** Burnout difference between the control group and the intervention group (N=50).

Burnout difference	Participants, n (%)	Values, mean (SD)	Values, SE
Control	25 (50)	0.3040 (2.08536)	0.41707
Web-based therapy	25 (50)	3.3480 (3.05070)	0.61014

### Reliability

Cronbach α was used to measure the internal reliability, where a Cronbach α of ≥.70 is considered acceptable, Cronbach α of ≥.80 is considered good, and an Cronbach α of ≥.90 is considered excellent. The coefficient α for international reliability showed good results (N=50; Cronbach α=.858; mean 1.826, SD 3.008; Cohen *d*=0.607; *P*<.001).

### Internal Validity Using Discriminant Statistics

The discriminant validity showed significant reliability and validity. Predicted group membership between the control group and the therapy group classifications was conducted on the pretest and posttest assessments for burnout and three EFP assessments—stress, motivation, and anxiety, as follows:

1. Pretest (78% of original grouped cases were correctly classified; effect size=0.733; Wilks lambda=0.711; *P*<.003). Subject matrix function pooled within-groups correlations between discriminating variable and standardized canonical discriminant functions the variable ordered by absolute size of correlation within function shows the following: stress pretest assessment <0.023, motivation pretest assessment >–0.281, anxiety posttest assessment >0.520, and burnout >0.423. Functions at group centroids functions shows the control group at –0.625 and the web-based therapy group at 0.625. There were no mission or out-of-range variables.

2. Posttest (70% of original grouped cases were correctly classified; effect size=0.682; Wilks lambda=0.784; *P*<.02). Subject Matrix function pooled within-groups correlations between discriminating variable and standardized canonical discriminant functions the variable ordered by absolute size of correlation within function shows the following: stress posttest assessment <0.814, motivation posttest assessment >–0.668, anxiety posttest assessment >0.495, and burnout >0.598. Functions at group centroids functions shows the control group at 0.514 and the web-based therapy group at –0.514. There were no mission or out-of-range variables.

### External Validity

An external construct validity question was asked after the pretest EFP and posttest EFP assessments. This study aimed to establish whether the measured results corresponded to how they felt at that point. All participants (the web-based therapy group and the control group) were asked to accurately validate (0-100) how they really felt in relation to the assessment results. All participants answered both validation questions, resulting in a (median=0.95 or >0.9414) construct validity for the pretest and a >0.9420 for the posttest (Table 3).

**Table 3:** Frequency table showing content validity results where each employee validates and confirms the accuracy of their assessed results to how they feel. A visual analog scale (VAS) is presented to the assessed employees, and they are prompted online to validate their scores. This is done during the web-based session with their counselor. The accuracy (validity) and correctness of the assessment measures for both preassessment for burnout (0.9414) and postassessment for burnout (0.9420) showed excellent content validity.

		Pretest burnout	Pretest accuracy	Posttest burnout	Posttest accuracy
**Participants, N**					
	Valid	50	50	50	50
	Missing	0	0	0	0
Values, mean (SD)		7.6240 (2.89972)	94.14 (5.322)	5.7980 (2.61249)	94.20 (5.379)
Values, median		8.0500	95.00	5.8000	95.00
Skewness		−0.179	−0.606	0.027	−0.590
SE of skewness		0.337	0.337	0.337	0.337
Kurtosis		−0.565	0.023	−1.057	−0.040
SE of kurtosis		0.662	0.662	0.662	0.662
**Percentiles**					
	25	5.5000	90.00	3.3250	90.00
	50	8.0500	95.00	5.8000	95.00
	75	9.9750	100.00	7.8000	100.00

## Discussion

### Principal Findings

The proverb “prevention is better than a cure” cannot be truer in the case of employee burnout. All individuals within this construct were affected by it. It is well proven that burnout can destroy a person’s ability to engage in work and life [4,14]. Burnout affects productivity within the workplace and directly affects company presenteeism rates [5]. Low productivity rates can also be correlated with employee defense routines [13,51].

Companies spend billions of US Dollars on preventive maintenance each year in efforts to maintain their physical machinery and equipment but very little on preventing unseen personnel burnout. Similar to machines that break when a part is not changed in time, productive employees experience burnout when their need to recharge is ignored [14,17,53]. In a *Harvard Business Review* article “1 in 5 Employees Is Highly Engaged and at the Risk of Burnout,” Seppäla and Moeller (2018 [54]) described how a company’s most important resource, their highly motivated employees, are at risk for burnout. These motivated employees are the ones that you repeatedly turn to, because they are dependable and efficient, and they always do the work. This affects work, colleagues, teamwork, innovation, creativity, and company continuity. Stress, anxiety, and psychosocial risks are widely recognized as major challenges to occupational health and safety, and these include stress, anxiety, depression, and burnout, which are all engagement killers that cost companies more than US $1 trillion annually [4,5,13,14,55].

As time and space continue to implode, companies increasingly depend on highly engaged employees. A company’s drive to increase output not only drives them to turn a blind eye to the needed or required employee maintenance but they also tend to ignore, all so frequent, signals by reframing those signals into resilience training to further increase the output of their highly engaged workers. Companies literally play Russian roulettes with the mental health of their most valued employees.

Addressing this problem requires the same discipline as your car service or any other scheduled maintenance. When an engine signal light comes on in your car, you take your car in for service. If you show signs of any risk for burnout, you should also take yourself in for tune-up or service. Preventive maintenance requires good tools to measure tolerances, deviations, and acceptable levels to operate sustainably. Unfortunately, today, there are many fancy-looking well-being or mental health apps on the market with 0 validity and reliability. Their flashy content looks good and they cost almost nothing in return, and as the old saying goes, “you get what you pay for” cannot be more from the truth. It is easy to provide a simple PDF one-page problem analysis without providing a solution or services to support companies in their preventive efforts is another whole story.

It has been shown that preventive and proactive treatment can reduce a person’s risk of burnout, and the chances that the differences occur through random error alone cannot be considered. This study found a statistically significant difference, showing that web-based therapy reduces the risk for burnout.

If used correctly, coaches, therapists, psychologists, and medical personnel can effectively reduce costs by proactively engaging with clients. The benefits of using the My-E-Health approach with the EFP batch mean that intervention can begin at an early phase when psychosocial challenges are in their infancy. This alone will reduce both the cost and effort needed to prevent the “slippery slope effect” into work-related burnout, as well as an individual’s personal pain.

Nevertheless, this full-service method can be used to proactively measure, identify, and treat other mental health challenges, such as anxiety, depression, and posttraumatic stress disorder. Therefore, broader use of the EFP batch as a measure of psychosocial situational well-being is highly plausible.

### Limitations

The study was registered in ClinicalTrials.gov on April 25, 2022, and not preregistered, as it was already approved by the Swedish Regional Ethics Committee in 2017; therefore, the authors do not see this as a limitation, but some might. Although there was an acceptable sample size of N=50 with an effect size of Cohen *d*=0.607, larger sample sizes are always desirable in clinical studies. In addition, published materials related to the prevention and rehabilitation of burnout are a limiting factor; however, this can be seen as an opportunity for clinicians and researchers to assess the clinical effectiveness of early identification and rehabilitation in future studies.

### Conclusions

Although there are many internet-based intervention studies on stress-related issues, few have addressed burnout. This original study suggests the effectiveness of web-based intervention therapy in reducing the impact of burnout in the workplace. It also provides good evidence for the use of web-based tools in prevention and early identification to measure company exposure and the risk of employee burnout. The EFP batch of web-based assessments can reliably assess company morbidity risk levels and successfully measure clinical interventions and rehabilitation, thereby reliably serving as both a diagnostic and therapeutic tool worthy of major research in the future.

Web-based therapy is effective in reducing burnout. Reliability of the burnout measure was good (Cronbach α=0.858; mean 1.826, SD 3.008; Cohen *d*=0.607; *P*<.001) as well as the instrument validity (0.9420). A paired samples test showed a good *t* score of 4.292 and *P*<.001, with a good effect size Cohen *d*=0.607.

Web-based therapy was effective in reducing the risk for burnout in participants compared with the control group. Tests of between-subject effects showed *F*=16.964 and a significant difference between the control group and the web-based therapy group: *P*<.001, with movement between the group variables or change of 0.261 or 26.1% for the dependent variable. Therefore, after controlling for pretest, is there a statistically significant difference between the levels of the independent variables?

## Abbreviations

ANCOVA: analysis of covariance
CBT: cognitive behavior therapy
EFP: empowerment for participation
EFPRB: EFP Risk for Burnout (extracted from the EFP assessment)
REST: Restricted Environmental Stimulation Technique
RR: relaxation response
VAS: visual analog scale

## Data Availability

The SPSS data file for this study is available as a supplementary file. Researchers can also contact the authors for related materials.
